# Optical Properties of CdSe/ZnS Nanocrystals

**DOI:** 10.6028/jres.119.026

**Published:** 2014-12-31

**Authors:** Adolfas K Gaigalas, Paul DeRose, Lili Wang, Yu-Zhong Zhang

**Affiliations:** 1 National Institute of Standards and Technology, Gaithersburg, MD 20899; 2 Life Technologies Corp., Eugene, OR 97402

**Keywords:** absorbance, CdSe/ZnS, fluorescence life time, fluorescence quantum yield

## Abstract

Measurements are presented of the absorbance, fluorescence emission, fluorescence quantum yield, and fluorescence lifetime of CdSe/ZnS nanocrystals, also known as quantum dots (QDs). The study included three groups of nanocrystals whose surfaces were either passivated with organic molecules, modified further with carboxyl groups, or conjugated with CD14 mouse anti-human antibodies. The surface modifications had observable effects on the optical properties of the nanocrystals. The oscillator strength (OS) of the band edge transition was about 1.0 for the nanocrystals emitting at 565 nm, 605 nm, and 655 nm. The OS could not be determined for QDs with emission at 700 nm and 800 nm. The fluorescence lifetimes varied from 26 ns for nanocrystals emitting near 600 nm to 150 ns for nanocrystals emitting near 800 nm. The quantum yield ranged between 0.4 and 0.9 for the nanocrystals in this study. A brightness index (*BI*) was used to evaluate the suitability of the nanocrystal labels for flow cytometer measurements. Most QD labels are at least as bright as fluorescein for applications in flow cytometer assays with 488 nm excitation. For optimal brightness the QDs should be excited with 405 nm light. We observed a strong dependence of the QD absorbance at 250 nm on the surface modification of the QD.

## 1. Introduction

The expression “QD” will be used in this work to describe CdSe/ZnS core/shell semiconductor nanocrystals which can be fabricated to yield specific optical properties [1]. An excellent review of QD structure and applications was given by Bera [2]. The application of QDs to multicolor flow cytometry has been described in depth by Chattopadhyay [3], and the suitability of QDs for quantitative measurements in flow cytometry has been discussed by Buranda [4]. This work examines the absorption and emission properties of several commercial QD fluorophores and presents the data in a manner suitable for estimating their utility for flow cytometer assays. An important part of the fabrication of QD is the passivation of the surface of QD with hydrophobic ligands, which have a significant effect on the optical properties [5], and facilitate further modification of the QD surface. For application in flow cytometry, the passivated surface of QDs is modified with coatings that induce water solubility and facilitate conjugation with antibodies to specific antigens found on the surface of human cells. When the conjugated QDs are incubated with cells, the antibodies on the QDs attach to the corresponding antigens on the surface of the cell. In a flow cytometer, the flow of a suspending liquid carries the cells past a focused laser beam which excites the fluorescence of the QDs attached to the cells. The fluorescence lasts during the transit time of the cell through the illuminating laser beam. In order to maximize the amount of the emitted fluorescence (for non-saturating illumination), the fluorescence quantum yield and the fluorescence absorbance have to be as large as possible [6]. In multiplexed assays, the cell surface has a variety of attached QDs, and the fluorescence emission, while the cell momentarily travels through the focal volume of the laser, contains the emission spectra from the different QDs. In order to maximize the accuracy of the interpretation of the fluorescence pulse, the emission spectra of different QDs need to have minimal spectral overlap so that efficient separation of the spectra can be achieved using optical filters. This work examines the absorption and emission properties of several commercial QDs fluorophores in order to quantify their performance in assays designed for multi-color flow cytometers.

## 2. Experimental Methods

### 2.1 Materials

The quantum dot materials measured were Qdot^®1^ probes obtained from Life Technologies™ corporation and used as received. The materials measured were Qdot^®^ ITK™ organic nanocrystals (organic-solution soluble), Qdot^®^ ITK™ Carboxy nanocrystals, and Qdot^®^ CD14 and CD8 mouse anti-human conjugates. The organic nanocrystals were suspended in decane solvent obtained from Sigma Aldrich (No 457116) and used without further purification. The phosphate buffer saline (PBS) was obtained from Life Technologies, and the Tween solutions were made by adding 0.02 % (by volume) Tween 20 detergent to PBS. The Qdot^®^ ITK™ Carboxy nanocrystals, and the Qdot^®^ CD14 and CD8 mouse anti-human conjugates were suspended in PBS or Tween solvents.

### 2.2 Absorbance

**Fig. 1:**
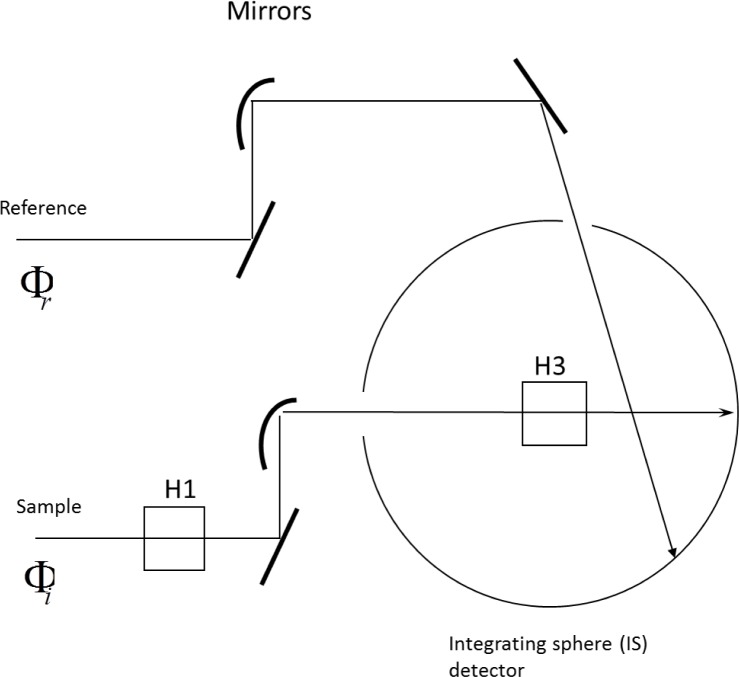
A schematic of the Perkin Elmer dual beam Lambda 850 spectrophotometer with an integrating sphere (IS) detector. The rectangle next to the number 1 represents the normal cuvette holder outside the integrating sphere (IS) detector, and the rectangle next to the number 3 represents the cuvette holder inside the IS detector. For both cuvette positions, the same reference beam enters the IS detector through a reference port and hits the wall of the IS detector. In practice, the same ‘auto zero’ spectrometer function is used for measurements in both cuvette holders. There are cuvette holders in the reference beam and in front of the IS sample beam entrance aperture; neither is shown in the diagram.

The absorbance measurements were performed with a Perkin Elmer Lambda 850 spectrometer equipped with a 150 mm integrating sphere (IS) detector. The IS detector permits a partial separation of signals due to absorption, scattering, and fluorescence. The layout of the cuvette sample holders in Lambda 850 spectrometer is shown in Fig. 1. The integrating sphere (IS) detector is a large sphere of diameter equal to 150 mm, with small holes for the entrance of the sample and reference light beams. Holder 1 (H1 in Fig. 1) is located outside the IS detector, and holder 3 (H3 in Fig. 1) is located inside the IS detector. The IS detector has a removable lid to facilitate the insertion and removal of holder 3 with the cuvette containing the sample. The procedure for combining the measurements in the two holders was described previously [7]. At a conceptual level, the relation between the measured absorbencies in the two holders and the sample properties is summarized in Eq. (1).

$$\begin{array}{l} A_{1}=N\sigma_{s}+N\sigma_{a}\\ A_{3}=N\sigma_{a}-N\sigma_{a}\Phi \end{array}$$ (1)

*A*_1_ and *A*_3_ are the measured absorbencies in holders 1 and 3, respectively. *N* is the number concentration in cm^−3^, σ*_a_* is the total absorption cross section in cm^2^, *σ_s_* is the cross section for scattering into angles outside the acceptance aperture of the instrument, and Φ is the fluorescence quantum yield. Implied in Eq. (1) is a 1 cm path through the sample. Fluorescence plays an important role in measurements with the sample placed inside the IS since the IS detector does not differentiate between the emitted fluorescence photons and the incident photons which are transmitted through the cuvette. Therefore, the measured absorbance, *A*_3_, inside the IS can be much smaller than the true absorbance. Scattering contribution is absent in *A*_3_ because in principle the IS detects all scattered photons. Since the diameter of individual QDs is much smaller than the wavelength of light, the scattering of light is almost isotropic, and the scattering cross section in Eq. (1) can be estimated by the total scattering cross section. Although scattering from individual QDs is expected to be small, there may be scattering from QD aggregates or/and impurities in the suspension. The scattering contribution in *A*_1_ was estimated by assuming that the absorbance for larger wavelengths (above the absorption peak edge in the spectrum) was due to scattering. If the absorbance *A*_1_ had constant value at these larger wavelengths, the constant value was subtracted from *A*_1_. Typically this constant value was less than 0.005 absorbance units (AU), and it was interpreted as scattering from larger impurities in the sample. Subsequent to the subtraction, any small wavelength dependency at the higher wavelengths was modeled using (*η*/*λ*)^4^ to describe scattering from small QD aggregates. Here *λ* is the wavelength in nm and the value of the parameter *η* was always less than 140. (The value of *η* depends on the fourth power of the particle radius and the square of the difference between the index of refraction of the particle and the surrounding medium.) The scattering contribution was also estimated using the difference *A*_1_ – *A*_3_ at larger wavelengths where fluorescence is absent and *A*_3_ should be due to absorption only, and the difference, *A*_1_ – *A*_3_, should be due to scattering only. The two estimates of the scattering contribution were consistent in all cases.

### 2.3 Fluorescence Emission

The steady-state fluorescence spectra were taken on a SPEX Fluorolog 3 (Jobin Yvon, Edison, NJ) spectrofluorometer using a continuous 450 W Xe lamp excitation source. A small fraction of the excitation beam was reflected, using a fused silica window, to a “reference” photodiode just before the sample to monitor the relative excitation intensity as a function of time and wavelength. The wavelength accuracy achieved over the entire wavelength range of the instrument was ± 0.2 nm for both emission and excitation, determined using atomic lamp standards. The relative radiometric accuracy as a function of wavelength of the reference (excitation) and signal (emission) detection systems was corrected using a calibrated detector and a calibrated light source, respectively, traceable to the NIST realization of the International System of Units (SI). All fluorescence measurements were taken between 22 °C and 24 °C using a 90 ° transmitting geometry.

In what follows, “fluorescence intensity” stands for the ratio of fluorescence signal to reference photodiode signal. The ratio corrects for signal intensity fluctuations due to changes in the excitation intensity with time. The “fluorescence intensity” emission spectra were corrected for the spectral responsivity of the detection system. A more detailed description of the qualification of the fluorescence spectrometer, related uncertainties and experimental conditions for certification and the determination of spectral correction factors is given elsewhere [8].

### 2.4 Fluorescence Lifetime

The life time of the fluorescence decay was measured using a LaserStrobe™ TM-30 time-resolved spectrofluorometer provided by Photon Technology International. The excitation wavelength was either the 337 nm output from the GL 3300 nitrogen laser or the 490 nm output from the GL 302 dye laser pumped by pulses from the GL 3300 nitrogen laser. In both cases the pulse duration was approximately 0.5 ns. In all cases, the instrument was run in the fluorescence decay mode and Istrobe configuration. The time delay from the excitation laser pulse to the start of detection cycle was set to 50 ns relative to the laser pulse. The nitrogen laser repetition rate was set to 10 Hz. The fluorescence signal at each time point was taken as the average of responses from five laser pulses

## 3. Results

### 3.1 Absorbance Measurements

**Fig. 2:**
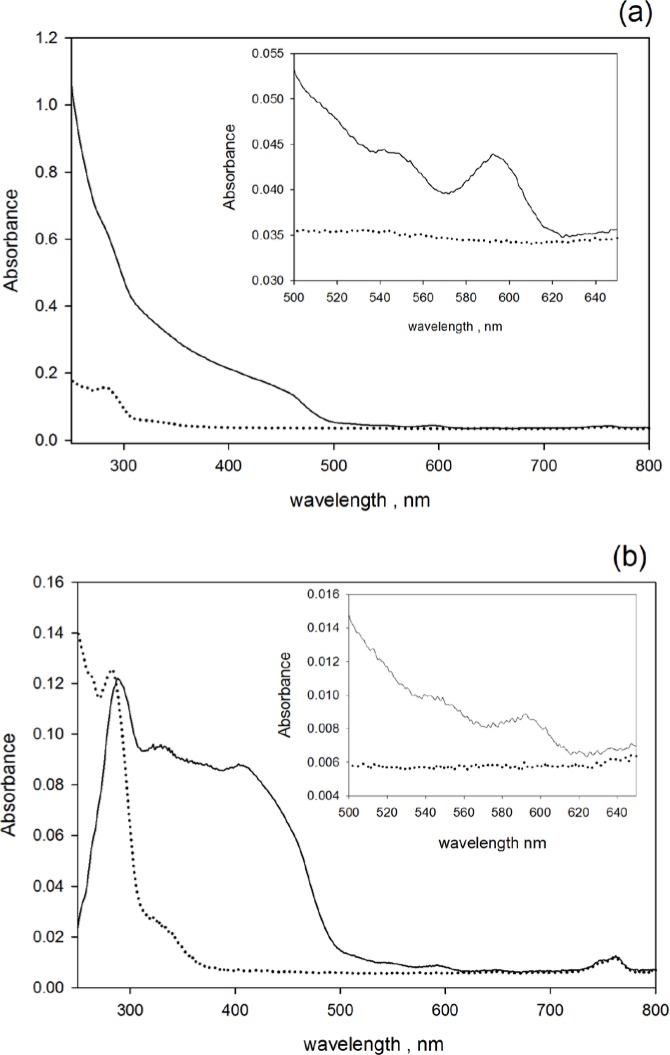
(a) The solid trace shows the measured absorbance of organic QD605 nanoparticles suspended in decane and placed in the cuvette holder outside the IS detector. The dotted trace shows the absorbance of “pure” decane. Most of the decane absorbance below 300 nm is due to aromatic impurities in commercially available decane. The inset in Fig. 2a shows an expanded portion of the absorbance spectra in the vicinity of the absorption peak at 595 nm. (b) The solid trace shows the measured absorbance of the same QD605 nanoparticles placed in the cuvette holder inside the IS detector. The dotted trace shows the absorbance of “pure” decane. The inset in Fig. 2b gives an expanded view of the region between 500 nm to 650 nm in the vicinity of the absorption peak at 590 nm. The striking difference between the measurements of absorbance outside and inside the IS detector is most likely due to the emitted fluorescence which is detected when the suspension is inside the IS. Subtracting the dotted trace (decane) from the solid trace (suspension + decane) in Fig. 2b leads to a large negative absorbance of the QD suspension. The negative absorbance is most likely due to the emission of multiple photons for each absorbed photon in the vicinity of 250 nm.

Absorbance measured in the holder outside and the holder inside the IS detector can be very different. The solid trace in Fig. 2a shows the measured absorbance of a suspension of organic QD605 in decane while the dotted trace in Fig. 2a shows the absorbance of the decane alone. (The notation QD605 refers to a Qdot^®^ with the emission peak at 605 nm). Both traces were taken with the cuvette placed outside the IS detector. The inset in Fig. 2a gives an expanded portion of the spectrum which shows the absorption peak at about 590 nm. The solid and dotted traces in Fig. 2b show the measured absorbances for the same QD sample and decane solvent with the cuvette placed inside the IS detector. The observed decane absorbance for wavelengths below 300 nm is most likely due to aromatic impurities in the commercial decane [9] which was used without further purification to dilute the QD stock suspensions. The solid traces in Fig. 2a and Fig. 2b are dramatically different. The trace in Fig. 2a is due to absorption and scattering by the QDs in the cuvette while the trace in Fig. 2b is due to absorption which is reduced by the emission of fluorescence. The IS detector is not sensitive to scattering, however the IS detector interpreted fluorescence emission as transmitted light and accordingly decreased the reported absorbance. Hence for fluorescent samples the measured absorbance inside the IS detector was always smaller than that measured outside the IS detector. Fluorescence is most likely the reason why the absorption peak is substantially reduced in Fig. 2b relative to the peak in Fig. 2a. The solid traces in Fig. 2a and Fig. 2b show a step-like increase in absorbance at about 500 nm. The step-like increase is more pronounced in the solid trace in Fig. 2b. A major difference between the two traces occurs for wavelengths below 350 nm. The absorbance measure outside the IS increases exponentially as the wavelength decreases while the absorbance measured inside the IS stays constant below 430 nm and then decreases sharply for wavelength less than 300 nm. Of special interest is the region around 250 nm where the absorbance shown by the solid trace in Fig. 2b decreases to values smaller than the absorbance of decane alone as shown by the dotted trace in Fig. 2b. Therefore for wavelengths around 250 nm, the amount of light that the IS detector interprets as “transmitted” is greater for the QD suspension than the decane solvent alone. Similar results were obtained for QD655. The measured absorbance inside the IS detector can be enhanced by re-absorption in the cuvette during the multiple reflections from the IS walls. This enhancement is expected for highly absorbing samples as is the case here for wavelengths below 300 nm. That is why the observed reduction in absorbance at 250 nm is surprising.

**Fig. 3:**
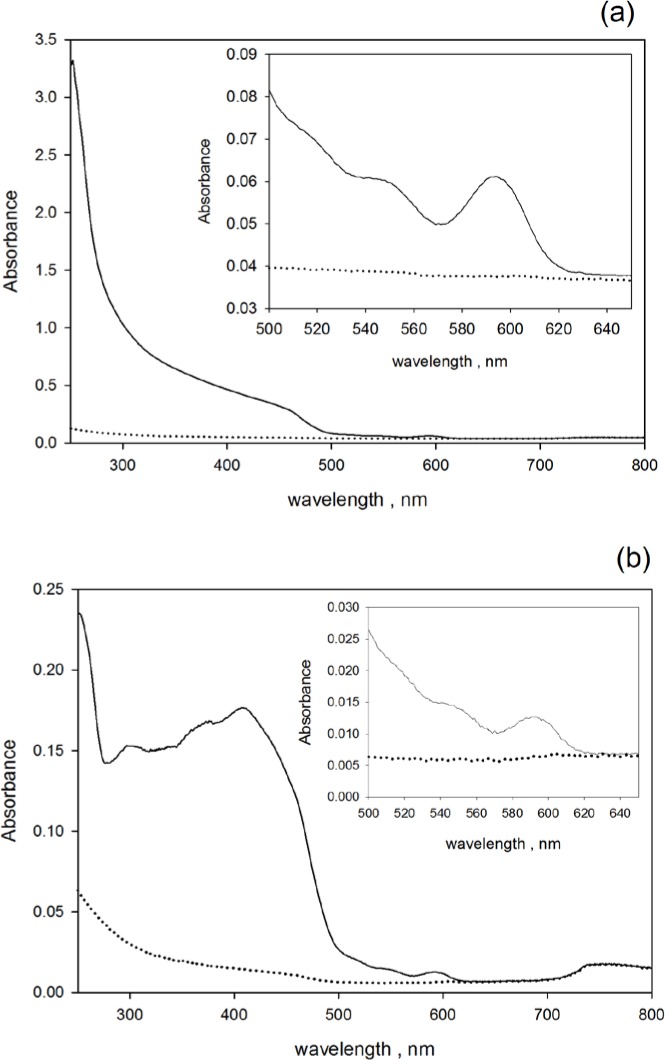
(a) The solid trace shows the measured absorbance of carboxylated QD605 nanoparticles suspended in deionized water and placed in the cuvette holder outside the IS detector. The dotted trace shows the absorbance of water. The inset in Fig. 3a shows an expanded portion of the absorbance spectra in the vicinity of the absorption peak at 595 nm. (b) The solid trace shows the measured absorbance of the same QD605 carboxylated nanoparticles placed in the cuvette holder inside the IS detector. The dotted trace shows the absorbance of water. The inset in Fig. 4b gives an expanded view of the region between 500 nm to 650 nm in the vicinity of the absorption peak at 595 nm. The striking difference between the measurements of absorbance outside and inside the IS detector is most likely due to the emitted fluorescence which is detected when the suspension is inside the IS. Subtracting the dotted trace (water) from the solid trace (suspension + water) in Fig. 3b leads to a positive absorbance of the QD suspension. The large spike in absorbance at 250 nm is most likely due to reabsorption of scattered photons. Unlike the case shown in Fig. 2b. there is no negative absorbance close to 250 nm.

Figure 3 shows the measured absorbance of QD605 with carboxylated surface suspended in PBS. The solid trace in Fig. 3a shows the measurement outside the IS detector, and the solid trace in Fig. 3b shows the measurement inside the IS detector. The inset in Fig. 3a and Fig. 3b shows an expanded view of the absorbance data in the wavelength region 500 nm to 640 nm. For all wavelengths, the absorbance measured outside the IS detector is much larger than the absorbance measured inside the IS. This difference results from the presence of fluorescence and scattering. Absorbance measured inside the IS detector is reduced by fluorescence, and the absorbance measured outside the IS detector is enhanced by scattering. In contrast to Fig. 2b, the absorbance given by the solid trace in Fig. 3b does not become less than the absorbance of the solvent. In fact the large peak in absorbance at 250 nm in Fig. 3b is most likely due to the absorption of light during the multiple reflections inside the sphere. For most QD samples in this study the measured absorbances were similar to those shown in Fig. 3a and Fig. 3b.

The dotted spectra shown in Fig. 2a, 2b and Fig. 3a, 3b were used to subtract the background absorbance from the sample absorbance prior to analysis. The analysis is discussed below. The two dotted traces in Fig. 2a and Fig. 2b, although with very similar dependence on wavelength, are offset on the vertical absorbance scale by an average value of 0.026. Measurements outside the IS detector are sensitive to the loss from reflections at cuvette surfaces while inside the IS detector, most of the light reflected from the cuvette surfaces falls on the walls of the integrating sphere and does not contribute to absorbance. Therefore the absorbance *A*_1_ is greater than the absorbance in *A*_3_ by the amount of light lost by reflection at the cuvette surfaces. In the present case, the difference should be about 0.031 absorbance units which is very close to the difference of values shown by the two dotted traces in the inset of Fig. 2a and Fig. 2b. Some of the light reflected from the cuvette surfaces will escape through the entrance aperture of the IS detector.

## 3.2 Fluorescence Emission Measurements

**Fig. 4:**
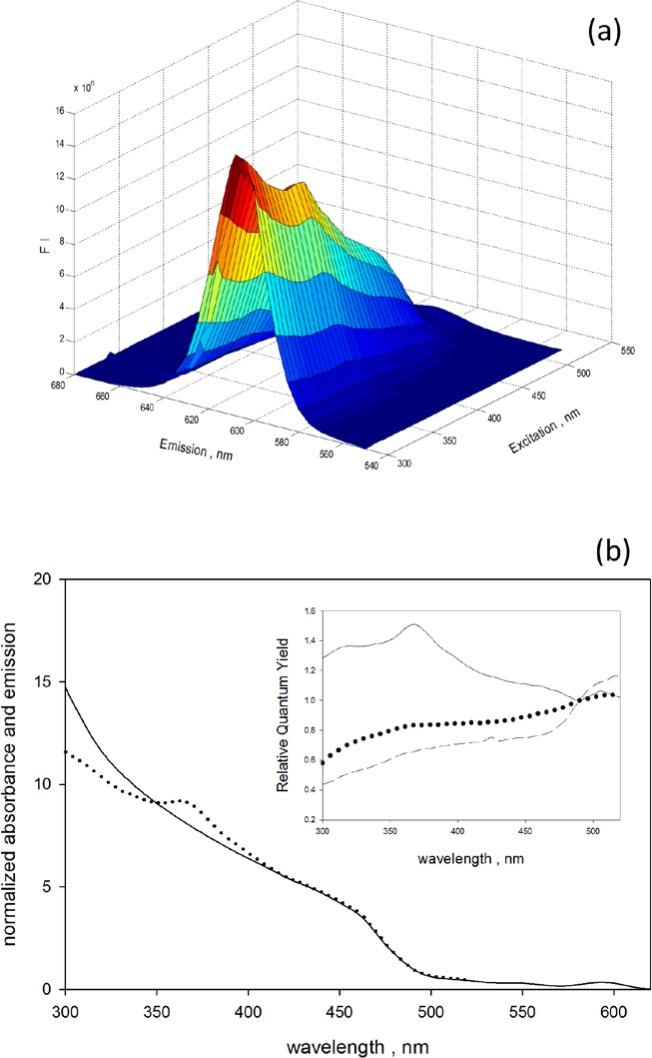
(a) Excitation-emission matrix for a suspension of QD605 with organic surfaces. The axis labeled “Excitation” gives the wavelength of the incident light which yields a fluorescence emission spectrum. The axis labeled “Emission” gives the fluorescence emission wavelength, and the axis FI gives the relative fluorescence intensity. (b) The dotted trace shows the integrated emission spectra as a function of the excitation wavelength. The fluorescence integrated emission spectrum was obtained by summing the emission spectrum in Fig. 4a at each excitation wavelength. The integrated emission spectra were normalized by the integrated emission spectrum at 490 nm. The solid trace in Fig. 4b gives the absorption cross section normalized by the value at 490 nm. In the inset of Fig. 4b, the solid, dotted, and dashed traces show the relative quantum yield (see Eq. (2) in text) obtained for suspensions of QD605, QD655, and QD800, respectively. In the case of QD605 (solid trace), the relative *QY* increases at shorter wavelengths. In the case of QD655 and QD800 the relative *QY* decreases significantly as the wavelength approaches 300 nm. The decrease is largest for QD800.

Figure 4a shows excitation-emission fluorescence spectra taken with the Fluorolog 3 spectrofluorometer of a suspension of QD605 (with organic surface) in a decane solvent. The excitation wavelength ranged from 300 nm to 520 nm and for each excitation wavelength an emission spectrum was collected for wavelengths between 550 nm and 680 nm. The emission spectra for each excitation wavelength in Fig. 4a were integrated and the results, normalized to the value of the integrated emission spectrum at 490 nm, are displayed by the dotted trace in Fig. 4b. The solid trace in Fig. 4b shows the absorbance measured for the same QD605 sample normalized by the absorbance at 490 nm. Both the normalized absorbance (solid trace) and the normalized fluorescence emission intensity (dotted trace) are small for wavelengths approaching the emission peak at 605 nm, and increase almost monotonically as the wavelength approaches 300 nm. The ratio of the normalized absorbance and the normalized fluorescence emission can be used to obtain the relative quantum yield as indicated in Eq. (2) [10].

$$\frac{\Phi_{x}}{\Phi_{ref}}=\frac{FI_{x}}{FI_{ref}}\frac{A_{ref}}{A_{x}}\frac{\lambda_{ref}}{\lambda_{x}}$$ (2)

The symbols Φ*_x_*, *A_x_*, *FI_x_* and Φ*_ref_*, *A_ref_*, *FI_ref_* stand for the quantum yield, absorbance and integrated fluorescence emission obtained with excitation wavelengths *λ_x_* and *λ_ref_*, respectively. The reference wavelength, *λ_ref_*, was set to 490 nm. The ratio of wavelengths in Eq. (2) converts the ratio of excitation energy fluxes into a ratio of photon number fluxes. The signal from the “reference” photodiode, which monitored the excitation intensity, was used to normalize the excitation energy flux to a constant value for all excitation wavelengths. The solid trace in the inset of Fig. 4b shows the fluorescence quantum yield at any excitation wavelength obtained with Eq. (2) relative to the fluorescence quantum yield at excitation wavelength of 490 nm. The dotted and dashed traces in the inset show the relative quantum yield obtained for suspensions of organic QD655 and QD800, respectively. In the case of QD605 (solid trace), the relative fluorescence quantum yield increase at smaller wavelengths, while for QD655 and QD800 the relative quantum yield decreases significantly as the wavelength approaches 300 nm. The decrease is largest for QD800. The smaller fluorescence quantum yield suggests that in the case of QD655 and QD800 a large fraction of the states excited by higher energy photons do not emit fluorescence. The dependence of the fluorescence quantum yield on excitation wavelength enters into the estimate of the relative signal from QD605, QD655, and QD800 when excited by light of the same wavelength, e.g., 405 nm.

### 3.3 Estimates of Absolute Quantum Yield

The excitation-emission matrix discussed above was used to obtain the quantum yield at any excitation wavelength relative to the quantum yield at the reference wavelength of 490 nm. In order to estimate the absolute brightness of a fluorophore, it is necessary to measure the absolute quantum yield at one wavelength, for example 490 nm.

The fluorescence quantum yield was measured using the technique described in Ref. [11]. A simplified version of Eq. (7) in [11] is presented below in Eq. (3). A typographic error in the original Eq. (7) is corrected in Eq. (3).

$$\begin{array}{l} \;QY=\frac{\left(10^{-A_{3}}-10^{-A_{1}}-r_{s}\right)}{(1-10^{-0.434\cdot a})t}\cdot \frac{E(\lambda )}{\lambda \cdot \langle E/\lambda_{f}\rangle }\\ \;A_{1}=A_{1}-\Delta_{scatt}\\ \;0.434\cdot a=A_{1}-A_{1}^{buf}\\ \;t=10^{-A_{1}^{buf}/2}\\ \;r_{s}=10^{-A_{3}^{buf}}-10^{-A_{1}^{buf}}\\ \langle E/\lambda_{f}\rangle =\int_{\lambda_{f}}\frac{E(\lambda_{f})}{\lambda_{f}}s(\lambda_{f})d\lambda_{f} \end{array}$$ (3)

**Fig. 5:**
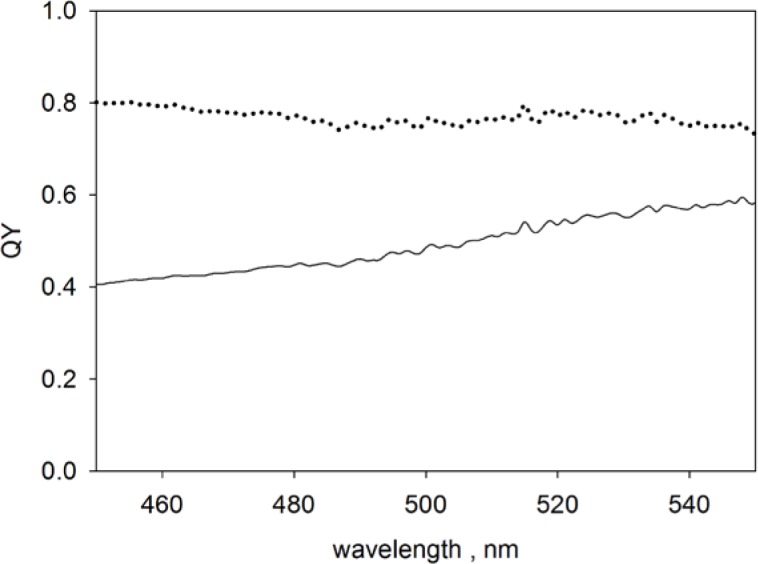
Solid trace shows the quantum yield (*QY*) obtained from the first factor in Eq. (3) for QD605 with organic surface. The dotted trace shows the final value of the *QY* obtained after the correction for detector efficiency.

The quantum yield (symbol of *QY* in Eq. (3)) depends on two factors, the first factor contains terms which depend on the measured absorbances inside (subscript 1) and outside (subscript 3) the IS detector, and the second factor contains a ratio of the detection efficiency at the excitation, *λ*, and emission, *λ_f_*, wavelengths. The first factor in Eq. (3) was evaluated using the absorbances shown in Fig. 2a and Fig. 2b where *A*_1_, *A*_3_ stand for the measured absorbances (without buffer subtraction) of the QD suspension placed in the holders outside and inside the IS detector, respectively. The second line in Eq. (3) includes the correction for scattering which was not included in the original formula. The scattering correction, which in all cases was less than 10 % of the total absorbance, is somewhat subjective. The second factor in Eq. (3) was evaluated using the normalized fluorescence emission spectrum of QD605 and the relative photomultiplier cathode radiant sensitivity given in Table 2 in [11]. The solid trace in Fig. 5 shows the *QY* of the organic-solvent soluble QD605 in decane evaluated using only the first factor in Eq. (3) and the dotted trace shows the *QY* evaluated using both factors in Eq. (3). The efficiency correction (second factor in Eq. (3)) is important because the fluorescence wavelengths and the excitation wavelengths may be very different. The mean quantum yield of QD605 in decane in the wavelength range 450 nm to 500 nm was 0.78 ± 0.06. The uncertainty is due mainly to the dependency of *QY* on excitation wavelength which in turn is mainly due to uncertainty in the relative spectral response.

In order to check the validity of the values of *QY* obtained with Eq. (3), the relative *QY* of QD605 modified with CD14 was measured using fluorescein as the reference fluorophore with a known quantum yield of 0.94. The relative *QY* was calculated using the expression in Eq. (2) which is valid for solutions with small absorbance. In this case, Φ*_x_* and Φ*_ref_* are the quantum yields of the QD and reference fluorophores, respectively. *A_x_* and *A_ref_* are the absorbances (with buffer subtracted) of the QD suspension and the reference fluorophore solution, respectively. *FI_x_* and *FI_ref_* are the fluorescence intensities of the QD and reference solution after spectral correction and conversion to photon flux. The ratio of the excitation wavelengths in Eq. (2) was set to 1 since the same excitation was used for both the QD suspension and the reference solution. The relative method gave *QY* = 0.77 ± 0.08, which can be compared with the value of 0.84 ± 0.06 obtained from Eq. (3). The two values of *QY* for QD605 modified with CD14 are within the expected combined uncertainty of the two methods. The values of *QY* obtained using Eq. (3) for a variety of QDs are shown in Table 1 in Sec. 4.4. The range of *QY* values in Table 1 is consistent with those reported by Bera [2].

### 3.4 Fluorescence Life Time Measurements

**Fig. 6:**
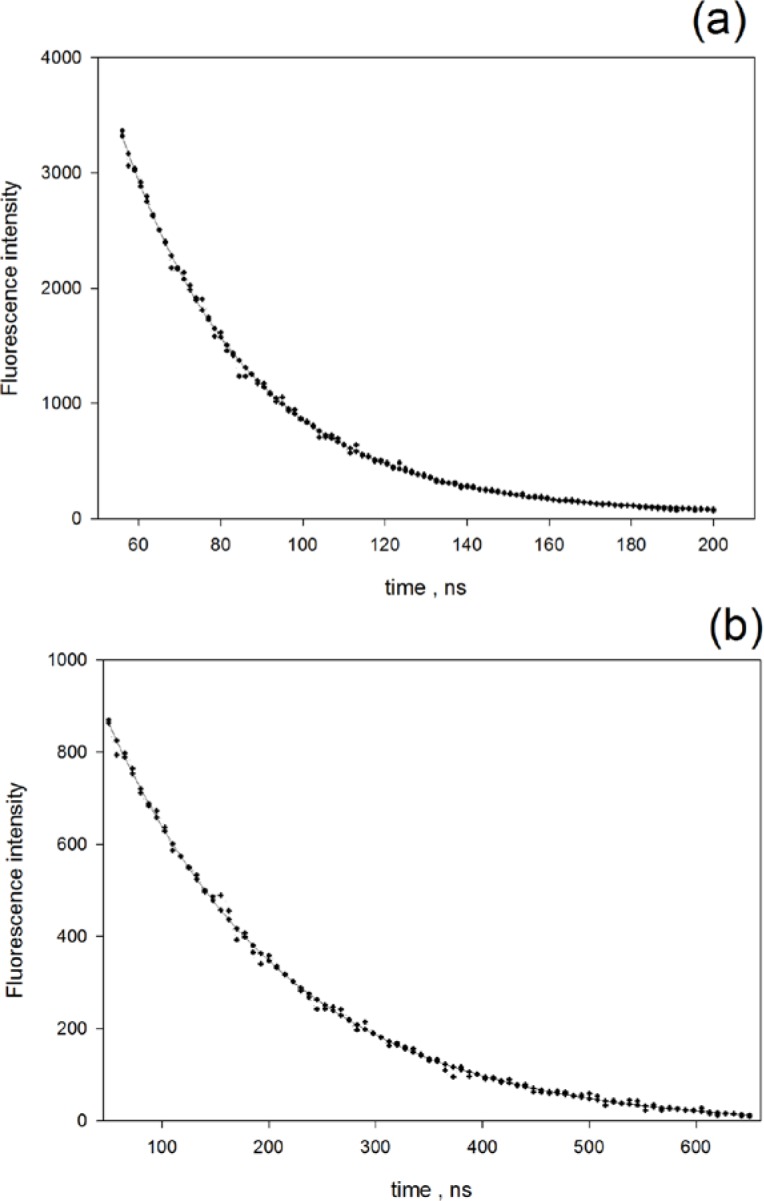
(a) The dotted trace shows the decay of the fluorescence emission of QD605 with organic surface excited with 337 nm light. The solid trace is the best fit to a single exponential function. (b) The dotted trace shows the decay of the fluorescence emission of QD800 with organic surface excited with 488 nm light. The solid trace is the best fit to a single exponential function.

The dot symbols in Fig. 6a show the decay of the fluorescence emission at 605 nm from a suspension of organic-solvent soluble QD605 in decane and excited with 337 nm light. The solid trace in Fig. 6a is the best fit to a single exponential function with a decay time of 31.5 ns. Examination of the data and fit points suggests that a single exponential is a good representation of the data. Similar results were obtained for QD655. The dot symbols in Fig. 6b show the decay of the fluorescence emission at 800 nm from a suspension of carboxylated QD800 excited with 490 nm light. The solid trace in Fig. 6b is the best fit to a single exponential function with a decay time of 168.4 ns. Again, the good fit to the data indicates that a single exponential gives a good representation of the data.

The fluorescence emission at 800 nm was not detected when the carboxylated QD800 suspension was illuminated with the 337 nm pulse from the nitrogen laser. Even with the detector delay set to coincide with the arrival of the pulse, there was minimal detected signal. With 490 nm illumination, the signal from the carboxylated QD800 was strong but was substantially weaker than the signal from carboxylated QD605 as expected from the reduced spectral response of the detector at higher wavelengths. The same behavior was also observed with the suspension of organic QD800. It is not clear why 337 nm pulses do not lead to observable fluorescence from QD800.

## 4. Discussion

### 4.1 Electronic Structure of CdSe/ZnS Core-Shell Nanocrystals

The optical absorption spectrum, photoluminescence (fluorescence), and life times depend on the nature of the electronic states of the CdSe/ZnS nanocrystal. The electronic states can be described on a conceptual level by considering the molecular orbitals which arise in the CdSe/ZnS material. The electronic configurations of isolated Cadmium (Cd) and Selenium (Se) atoms are (core) 4d^10^5s^2^ and (core) 4s^2^ 4p^4^, respectively, where the symbols (core) stands for the electronic configurations of the tightly bound electrons which are assumed not to interact with neighboring atoms. The above configurations are based on the energies of the atomic orbitals of Cd and Se which have been calculated using Hartree-Fock method [12]. Other orbitals are not included because their energies are much lower and presumably they have a minor effect of the properties of the nanocrystal. The crystal structure of CdSe is wurtzite (hexagonal close packed, hcp) with each Cd atom surrounded by four Se atoms, and each Se atom surrounded by four Cd atoms [13]. Given the location of each nucleus and the associated atomic orbitals, it is possible to perform calculation of the electronic structure of the nanocrystal. Such a calculation has been done for CdSe nanocrystals capped with formic acid [14]. The calculation showed that the low energy absorption peak was associated with band edge transitions between molecular orbitals composed mainly of Se 4p atomic orbitals (HOMO) to molecular orbitals composed mainly of Cd 5s atomic orbitals (LUMO). In both cases, the molecular orbitals were delocalized over many atomic sites. In the case of a CdSe capped with a ZnS shell, one would also expect electronic transitions from molecular orbitals composed of S 3p atomic orbitals to molecular orbitals composed of Zn 4s atomic orbitals. The energy of these transitions is expected to be higher than in the case of the CdSe core. It may be that the step-like increase in absorbance observed around 500 nm in almost all Qdot suspensions is associated with these transitions.

The CdSe/ZnS nanoparticle composed of 20 Cd, 20 Se, 30 Zn, and 30 S atoms would have about 1100 molecular orbitals originating from the basis states discussed previously. Some of these molecular orbitals could be involved in higher energy electronic transitions, however they could not account for the exponential increase in the observed absorption cross section below 300 nm. Deeper atomic orbitals would provide a large number of additional molecular orbitals, however the expected energies of these molecular orbitals would be much larger than the maximum observed energy of 5 eV (250 nm). Therefore the large increase in absorption below 300 nm is most likely due to factors other than the available molecular states in the CdSe/ZnS nanocrystal.

In summary, the absorbance of the QD suspension is consistent with the existence of a band gap transition at low energy associated with the CdSe core, an onset of transitions in the ZnS cladding leading to the “step” in the absorbance, and an array of optical transitions at higher energies which are likely associated with the surface states. The fluorescence of the QDs is characterized by a single peak which has been interpreted as the optical relaxation of the delocalized states on the band edges (LUMO to HOMO) in the CdSe core. This suggests that the excited delocalized states relax rapidly to the lowest delocalized excited state which has a comparatively slow radiative relaxation. The above discussion will provide a framework for the discussion of the measurements below.

### 4.2 Estimation of Oscillator Strength (OS)

The measured QD absorbance, *A* = (*A*_1_−*A_buf_*), was converted to an absorption cross section *σ_abs_* in m^2^, using Eq. (4).

$$\sigma_{abs}=\frac{2.303\cdot A\cdot 1000}{N_{A}\cdot C}\cdot 10^{-4}$$ (4)

**Fig. 7:**
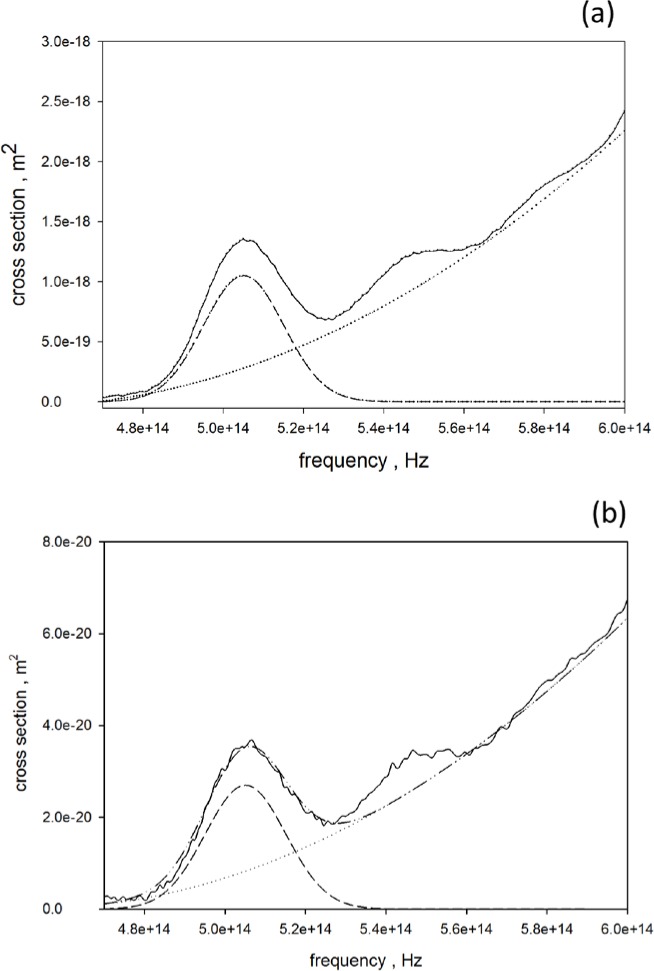
(a) Solid trace shows the measured absorption cross section as a function of frequency for QD605 with organic surface. The dashed and dotted traces show the Gaussian peak and a quadratic polynomial which best fit the observed cross section. The area of the Gaussian peak was integrated to obtain the oscillator strength as described in the text. (b) Solid trace shows the measured absorption cross section as a function of frequency for QD605 with carboxylated surface. The dashed and dotted traces show the Gaussian peak and a quadratic polynomial which best fit the observed cross section.

The concentration, *C*, mol/L, of the QD sample was estimated by multiplying the value of the stock suspension given by the manufacturer by a dilution factor. As an example, 20 µL of the stock suspension of carboxylated QD605 with a concentration of 8 µmol/L was placed in 3mL of PBS buffer giving a sample concentration, *C*, of 0.053 µmol/L. The solid trace in Fig. 7a, reproduces the converted absorbance data from Fig. 3a, and shows the absorption cross section of carboxylated QD605 in units of m^2^ as a function of frequency (obtained from the wavelength in nm using *ν* = 3·10^17^/*λ*). The solid trace in Fig. 7b shows the converted absorbance data for organic QD605 taken from Fig. 2a. In both Fig. 7a and Fig. 7b, the dotted and dashed traces show the background and Gaussian peak, respectively, which best reproduce the data shown by the solid trace. The cross sections shown in Fig. 7a and Fig. 7b have similar shapes, however at higher frequencies the carboxylated QD605 cross section increases significantly more with increasing frequency (not shown in Fig. 7). The oscillator strength, *f*, was calculated using the expression given by Eq. (5) [15, 16].

$$f=\frac{4\cdot m\cdot \varepsilon_{0}\cdot c}{e^{2}}\int \sigma (\nu )d\nu$$ (5)

The symbol *m* stands for the electron mass (kg), *ε*_0_ is the free space permittivity, *c* is the speed of light (m/s), *e* is the electron charge (C), and *σ* is the cross section (m^2^). The Gaussian peaks in Fig. 7a and Fig. 7b were integrated over a frequency interval whose bounds were selected to give vanishingly small values of the peak function at the boundaries. For carboxylated QD605, *f* = 1.14 (Fig. 7a) and for organic QD605, *f* = 0.99 (Fig. 7b). These values of the oscillator strength are consistent with the presence of several single electron transitions in the observed absorbance peak. Model calculations [14] show that, for the transitions near the band edge, the value of the oscillator strength is about 0.15 for each transition, and that three to four single electronics transitions are located in the observed absorption peak. The radiative lifetimes corresponding to these oscillator strength values are 4.8 ns and 5.5 ns for the carboxylated QD605 and organic QD605, respectively. The expected lifetimes are smaller than the observed values of 40.7 ns and 31.5 nm for the carboxylated QD605 and organic QD605, respectively, excited with 347 nm light. The large radiative lifetimes suggest that the direct transition from the excited electronic state to the ground state is hindered. Observations on single CdSe QDs have shown that the lifetime can switch between several values associated with different excited states [17]. The fact that the observed decay fits a single exponential suggests that the excited states participating in the radiative decay are of similar character. The fact that the radiative decay may contain several electronic transitions has bearing on the width of the observed fluorescence peak. The width of the peak impacts the accuracy of the compensation corrections in multicolor flow cytometry measurements.

### 4.3 Absorbance at 250 nm

The optical properties of QD at 250 nm are not useful for practical flow cytometer measurements. However these properties may be of utility as diagnostic tools during manufacture process of QDs.

**Fig. 8:**
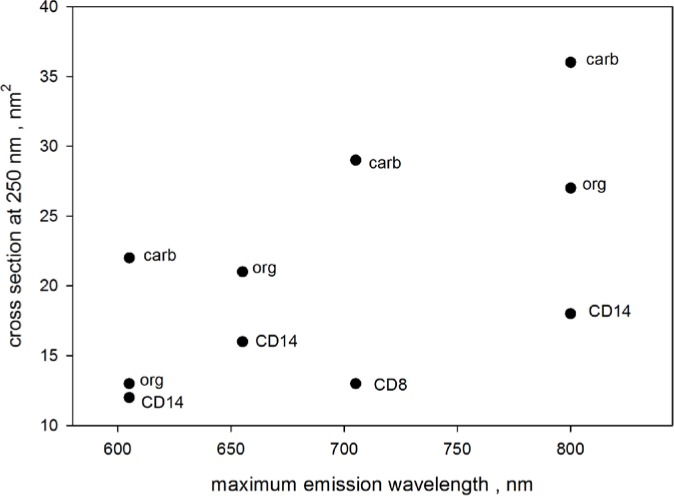
Absorption cross section measured at 250 nm for QDs with different diameters and surface properties. The horizontal axis gives the wavelength of peak fluorescence emission which is known to correlate with diameter. The vertical axis gives the measured cross section at 250 nm. Each QD with a specific emission had three surface properties: organic (org), carboxylated (carb), and functionalized with CD14 or CD8. The absorption cross section depended on the type of surface. The uncertainties are of the order of two diameters of the black circles.

During the analysis of QD absorbance, it became evident that the QD absorbance measured outside the IS detector at 250 nm exhibited a dependence on surface modification. Fig. 8 shows the measured absorption cross section at 250 nm for four different QD suspensions emitting at 605 nm, 655 nm, 705 nm, and 800 nm. The QD in each population had surfaces that were organic (“org” in Fig. 8), carboxylated (“carb” in Fig. 8) or with antibodies (“CD” in Fig. 8). The cross sections at 250 nm are highest for QDs with carboxylated surfaces, and lowest for QDs with CD surfaces. Wherever observed, cross sections for QDs with organic surfaces fall in between. This pattern was maintained for all QDs studied, and the existence of this pattern was well outside uncertainty which is about two diameters of the black circles in Fig. 8. The uncertainty was estimated from the uncertainty of the measured absorbance, the uncertainty in the scattering contribution, and the uncertainty in the concentration of QD. A possible interpretation of the QD absorption at 250 nm is that it is due to transitions involving charge transfer to solvent (surface) states (CTSS). CTSS has been invoked to explain the strong temperature dependence of absorption of solutions of halogen ions (Cl^−^, I^−^) near 200 nm [18].

**Fig. 9:**
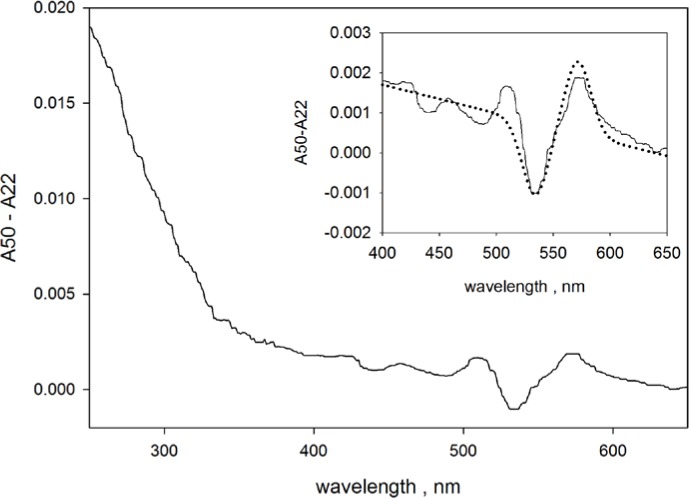
Temperature dependence of the absorbance of carboxylated QD565 in PBS. The solid trace shows the difference in the absorbance measured at 50 °C and 20 °C. The inset in Fig. 9 gives an expanded view of the region around 550 nm. The difference at 550 nm is typical response due to the shift in the wavelength of maximum emission. The dotted trace in the inset gives the expected response for a Gaussian peak which shifts to the red at higher temperatures. The response at 250 nm is a change in amplitude with the absorbance increasing by about 3 % at the higher temperature.

In addition to the dependence on surface modification, the QD absorbance at 250 nm also depends on temperature. As an example of this dependence, absorbance measurements were performed on suspensions of carboxylated QD565 at 22 °C and 50 °C. Both absorbance spectra had the appearance of those shown in Fig. 3a. The solid trace in Fig. 9 show the difference in the absorbance measured at 50 °C and 22 °C (*A*(*T* = 50 °C)-*A*(*T* = 22 °C)). The difference in PBS absorbance measured at 22 °C and 50 °C is approximately 0.0007 for wavelengths between 250 nm and 300 nm. The subtraction of the two QD spectra automatically subtracted out the PBS buffer contribution. The solid trace in the inset on Fig. 9 shows an expanded region around the absorption band at approximately 550 nm. The dotted trace in the insert of Fig. 9 shows the difference of two Gaussian peaks superimposed on a slightly sloping linear background. The amplitudes of the two Gaussian peaks are equal but their maxima are displaced by 38 nm. The comparison of the solid and dotted traces in the inset of Fig. 9 suggests an interpretation of the observed data (solid trace) as a shift of the peak to the red at increased temperatures with the peak amplitude remaining unchanged. However, the expected red-shift of the absorption peak is about 6 nm [19] so that the above interpretation has to be modified. In contrast to the absorbance at 565 nm, the absorbance at 250 nm increases by about 3 % at 50 °C relative to the value at 22 °C. This is a significant change which contrasts with relative constancy of the absorption peak at 565 nm. The CTSS spectra have been observed to shift to higher wavelengths at increased temperatures [20].

The absorbance at 250 nm measured inside the IS detector became negative for some QD samples (see the difference between the solid and dotted traces in Fig. 2b). This is most likely due to carrier multiplication in the nanocrystal core [21]. Multiplication occurs when a highly excited electron-hole state relaxes by exciting a lower energy electron-hole state. The result is two electron-hole excited states which can equilibrate sequentially to a common lowest energy excited state which decays radiatively. The result is the emission of two fluorescence photons. Emission of two low energy photons (605 nm) after the absorption of a high energy photon (250 nm) could lead to an apparent negative absorbance.

The occurrence of transitions to surface states and carrier multiplication complicates the interpretation of the QD absorbance at 250 nm. However, this wavelength region may provide an opportunity to gain new insight into the photophysical behavior of QDs, as well as develop new tools for monitoring the production of QDs.

### 4.4 Brightness Index

The Qdots studied in this work are used to label receptors on the surface of lymphocytes and other biological cells. The number of receptors on the cell is determined by illuminating the cell with light and measuring the emitted fluorescence. For best signal to noise ratio, it is very important to maximize the number of emitted fluorescent photons. In the case of a flow cytometer, the relative intensity of the fluorescence signal produced by a fluorophore can be estimated by the fluorophore’s brightness index, *BI*, which is defined in Eq. (6) below (also see Appendix).

$$BI=\sigma_{abs}\cdot QY$$ (6)

As discussed in the Appendix, if the decay rate (given by 1/*τ*) is much greater than the absorption rate (given by *σ_abs_I*) then the fluorescence intensity is given by the product of *BI* and the incident energy flux. This condition is met for most commercial flow cytometers and the fluorophores shown in Table 1. The values of *BI* for the QDs studied in this work are given in Table 1. Fluorescein is included in Table 1 as a reference fluorophore. Table 1 does not include *QY* values for QD800 because a reliable spectral response correction for the apparatus was not available at red wavelengths. (The *QY* measurements utilized the generic spectral response of the photomultiplier detector provided by the photomultiplier manufacturer. The spectral response at 800 nm is almost an order of magnitude less than at 450 nm, and the correction at 800 nm has to be determined for the specific PMT used in the apparatus.) The *QY* values for QD705 in Table 1 may also be biased by the uncertainty in the spectral correction. The values of *QY* in Table 1 were obtained using Eq. (3) and were reasonably constant in the range of wavelengths between 450 nm and 500 nm, and therefore these values of *QY* were also used to estimate the *BI* at 405 nm in Table 1.

A better estimate of *BI* at 405 nm could be obtained by using the measured relative *QY* shown in Fig. 4b. The relative *QY* was measured for only three QDs and the improved *BI* estimates differed by no more than 20 % form the values in Table 1. The data in Table 1 suggests that QD605 (CD14) would be an excellent label for excitation with 405 nm; even with 488 nm excitation QD605 would be superior to fluorescein. The label QD565 is a poor label when excited with 488 nm, however it becomes a much better label when excited with 405 nm laser. A table similar to Table 1 could be produced for other laser wavelengths used in flow cytometers. Table 1 does not include reduction of the fluorescence intensity due to “blinking”. However, recent advances in the fabrication of QDs have suppressed blinking to the point where it may be neglected [22].

*BI* serves as a good indicator of label performance for flow cytometers because photodegradation can be neglected. For measurement with microscopes, the illumination time can be several seconds and photo degradation has to be included in the calculation of Brightness Index. Fluorescein intensity is known to decrease rapidly with extended illumination, while QDs are known to be less susceptible to photodegradtion. QDs may be the fluorophore of choice for imaging applications.

## 5. Conclusion

The utility of the QD fluorophores in flow cytometer applications is expected to be as good as or better than fluorescein when excited with a 488 nm laser. A major advantage of QDs is that a single excitation wavelength (e.g., 405 nm) can be used to excite a large number of QDs emitting at different wavelengths corresponding to different fluorescence channels in a flow cytometer. The width of the emission peak of QD is still broad so that cross talk between different fluorescence channels in a flow cytometer cannot be neglected. In principle, all of the QDs studies in this work can be used in flow cytometer applications. The photo physical measurements presented in this work provide a basis for estimating the fluorescence signal in these applications. In practice, most of the QDs have not been used extensively in flow cytometer measurements for reasons such as unfamiliarity, toxicity concerns, and general inertia to change. Significant progress is expected in the development of semiconductor nanocrystals so that use of QDs as biological labels is expected to increase [23].

## Figures and Tables

**Table 1:** Brightness Index, *BI* = *σ_abs_*·*QY*·10^18^

Fluorophore	*σ_abs_*(488) 10^−18^ m^2^	*σ_abs_* (405) 10^−18^ m^2^	Lifetime *τ*, 10^−9^ s	QY	*BI* (488)	*BI* (405)
Fluorescein^1^	0.031		4.3	0.94	0.03	–
QD605 (organic)^3^	0.43	2.62	31.5	0.78±0.06	0.34	2.04
QD655 (organic)^3^	1.27	4.14	33.4	0.73±0.07	0.93	3.02
QD800 (organic)^3^	1.48	4.57	150			
QD565 (carboxyl)^4^	0.13	0.43	26.2	0.43±0.05	0.06	0.18
QD605 (carboxyl)^1^	0.52	2.82	40.7	0.74±0.04	0.38	2.09
QD705 (carboxyl)^1^	1.31	3.87	131	0.30±0.04	0.39	1.16
QD800 (carboxyl)^4^	1.85	4.95	168			
QD605 (CD14)^1^	0.67	2.87	43.1±0.05	0.84±0.06	0.56	2.41
QD605 (CD14)^2^	0.90	3.03		0.85±0.08	0.77	2.58
QD605 (CD14)^1^	0.89	3.13		0.87±0.06	0.77	2.72
QD655 (CD14)^2^	1.21	3.19	41.6±0.04	0.50±0.04	0.61	1.60
QD705 (CD8)^4^	1.22	2.90	126±4	0.43±0.06	0.52	1.25
QD800 (CD14)^4^	1.56	3.68	160±2			

1 PBS

2 PBS+TWEEN

3 decane

4 distilled water

cross section uncertainty about 10 %, life time uncertainty about 6 %

## 6. Appendix

The fluorescence emitted by a fluorophore can be modeled using a two state system. The equilibration process in the excited state is assumed to be instantaneous and will be neglected. Let *N*_0_ and *N*_1_ be the populations of the ground state and the excited state, respectively. In addition it will be assumed that the total number of fluorophores, *N*, is constant and no photo-bleaching occurs. The time evolution of the population of the excited state is given by Eq. (A1).

$$\frac{dN_{1}}{dt}=k_{a}N_{0}-k_{r}N_{1}=k_{a}(N-N_{1})-(k_{f}+k_{nr})N_{1}$$ (A1)

The absorption rate is given by *k_a_* = *σI* where *σ* is the photon absorption cross section and *I* is the photon flux in number per area per second. The relaxation rate of the exited state is given by *k_r_* = *k_f_* = *k_nr_* = 1/*τ* where *k_f_* is the radiative decay rate, *k_nr_* is the non-radiative decay rate, and *τ* is the life time of the excited state. Assuming continuous illumination, the equilibrium population of the excited state can be found from Eq. (A1) by setting the derivative *dN*_1_/*dt* = 0. After some algebra, the resulting equilibrium fluorescence intensity per fluorophore, *I_f_* = *k_f_ N*_1_/*N*, is given by Eq. (A2).

$$I_{f}=\frac{k_{f}N_{1}}{N}=\frac{\Phi \sigma }{\tau }\frac{I}{\left(k_{a}+k_{r}\right)}=\frac{\Phi \sigma }{\tau }\frac{I}{\left(\sigma I+1/\tau \right)}$$ (A2)

Where the fluorescence quantum yield, Φ, is given by Φ = *k_f_* /(*k_f_* + *k_nr_*) = *k_f_ τ*. Equation (A2) shows that *I_f_* saturates at large illumination intensities and depends linearly on the illumination intensity at small values of *I*. This behavior was discussed previously by Engh [4]. Most flow cytometers have lasers with power output of about 40 mW. Focusing the laser beam to an area with a radius of 1·10^−6^ m, gives an energy flux of 10^10^ W/m^2^ at the focal point. At this energy flux, the decay rate is much greater than the absorption rate and the fluorescence intensity in Eq. (A2) is equal to Φ*σI*. Therefore the product of the quantum yield and the absorption cross section characterizes the expected fluorescence emission of a fluorophore in the flow cytometer.
